# Mesorectal shape variation in rectal cancer radiotherapy in prone position using a belly board

**DOI:** 10.1016/j.phro.2021.08.001

**Published:** 2021-08-22

**Authors:** Maurice C. Cox, Pètra M. Braam, Heidi Rütten, Ruud van Leeuwen, Markus Wendling

**Affiliations:** Department of Radiation Oncology, Radboud University Medical Center, Geert Grooteplein Zuid 32, 6525 GA Nijmegen, the Netherlands

**Keywords:** Rectal cancer, Radiotherapy, PTV margin, Belly board, Cone-beam CT

## Abstract

**Background and purpose:**

In rectal cancer patients, radiotherapy in prone position using a belly board can reduce the dose to organs at risk. For this patient group we investigated inter-fraction shape variation of the mesorectal part of the clinical target volume (CTV) and determined planning target volume (PTV) margins.

**Materials and methods:**

Patients with rectal cancer receiving neoadjuvant (chemo)radiotherapy were eligible. For each patient a planning computed tomography (pCT) and five cone-beam CT (CBCT) scans were acquired in prone position using a belly board. The mesorectal CTV was delineated on all scans. Mesorectal shape variation was quantified relative to the pCT. PTV margins were derived locally and averaged for separate subregions of the mesorectal CTV. For each patient a total PTV was constructed using our clinical margins for mesorectal and lymph node CTVs. An artificial dose distribution conforming to this PTV was used to calculate the coverage for the mesorectal CTV using the CBCT delineations.

**Results:**

In 19 rectal cancer patients the derived PTV margins were smallest in the upper-lateral region (6 mm) and largest in the upper-anterior region (16 mm). PTV margins for the upper-anterior region were larger for female patients (19 mm) compared to male patients (14 mm). Clinical margins for the total PTV were sufficient for a coverage of at least 97% of the mesorectal CTV for all patients.

**Conclusions:**

Mesorectal shape variation is heterogeneous and largest in the upper-anterior region, in rectal cancer patients irradiated in prone position and using a belly board.

## Highlights

• Mesorectal shape variation is diverse and largest in the upper-anterior region.
• Derived planning target volume margins for the upper-anterior region were larger in female patients.
• Planning target volume margins are comparable for radiotherapy and chemoradiotherapy groups.

## 1 Introduction

The standard of surgical resection for rectal cancer consists of total mesorectal excision [1], [2], [3]. Neoadjuvant radiotherapy (RT) is added for primary resectable disease with lymph node involvement or extramural invasion >5 mm, resulting in a lower local recurrence rate compared to surgery alone [4]. Examples of indications for neoadjuvant chemoradiotherapy (CRT) in locally advanced rectal carcinoma are invasion of other organs and structures (T4), or T3 with distance to the mesorectal fascia (MRF) < 1 mm [5], [6]. The most important organs at risk are the small bowel and colon (i.e., bowel bag). Multiple studies have shown a relationship between the dose to the bowel bag and the incidence of intestinal toxicity, such as acute radiation enteritis, chronic diarrhea, and less frequently bowel stricture, perforation and hemorrhage [7], [8]. Bladder filling protocols, prone positioning, and belly boards have been used to reduce the volume of bowel in the high-dose region by pushing the bowel bag away from the target volume [9], [10].

In order to reduce the dose to the organs at risk without decreasing the target coverage, advanced irradiation techniques such as volumetric modulated arc therapy (VMAT) have been developed [11], [12]. When performing RT with highly conformal VMAT plans, it is important to use optimal clinical target volume (CTV) to planning target volume (PTV) margins. These margins account for uncertainties in patient setup and organ motion/shape variation. In the mesorectal part of the CTV, shape variation is known to be substantial and heterogeneous [13], [14]. In previous studies a large variety of required PTV margins was suggested, especially in the upper region of the mesorectum, ranging from 7 to 31 mm in anterior direction [13], [14], [15], [16], [17], [18], [19], [20]. However, to our knowledge, PTV margins larger than 20 mm in clinical practice is uncommon in most treatment centers. PTV margins described in literature are mainly based on studies in which patients were irradiated either in supine or prone position without the use of a belly board [14], [16], [17], [18], [19], [20].

The purpose of this study was to investigate the inter-fraction shape variation of the mesorectum and determine required PTV margins in rectal cancer patients irradiated in prone position using a belly board.

## 2 Materials and methods

### 2.1 Patients, scans and treatments

A total of 20 patients (ten neoadjuvant RT and ten neoadjuvant CRT patients, five male and five female each) treated between June 2017 and October 2018 were retrospectively selected for this institutional review board exempt study. One patient was excluded from the analysis because of excessive scatter artefacts on cone-beam CT (CBCT). Baseline patient and treatment characteristics are listed in Table 1. Male and female patient groups were comparable regarding tumor location, TNM stage, MRF involvement and age at diagnosis (data not shown). For one of the remaining 19 patients, one CBCT scan was missing.

**Table 1:** Patient and treatment characteristics.

Sex (n)	
Male	10
Female	9
Age at diagnosis (years)	
Median	65
Range	39–88
Treatment (n)	
5x5 Gy male	5
5x5 Gy female	5
25x2 Gy male	5
25x2 Gy female	4
T-stage (n)	
T1	0
T2	1
T3	16
T3-4	1
T4	1
N-stage (n)	
N0	1
N1	7
N1-2	1
N2	10
M-stage (n)	
M0	17
M1	2
MRF involvement (n)	
No	10
Yes	9
Tumor location (n)	
Proximal (0–5 cm)	6
Mid-rectal (5–10 cm)	8
Distal (10–15 cm)	5

A computed tomography (CT) scan with 3 mm slice spacing was obtained for treatment planning, ranging from the L2–L3 junction to below the perineum. No intravenous, oral or rectal contrast was used. Patients were positioned in prone position using a belly board (MacroMedics, Waddinxveen, The Netherlands). For all patients a kilovoltage-CBCT scan was acquired before each fraction. For neoadjuvant RT patients (5×5 Gy) all CBCT scans were collected. For neoadjuvant CRT patients (25×2 Gy) five out of 25 CBCT scans were collected, one per week, randomly. All patients received instructions to drink 500 ml of water one hour before the planning CT (pCT) scan and every fraction, according to our full bladder protocol. No protocol regarding rectal filling/emptying was used.

### 2.2 Delineations

**Fig. 1:**
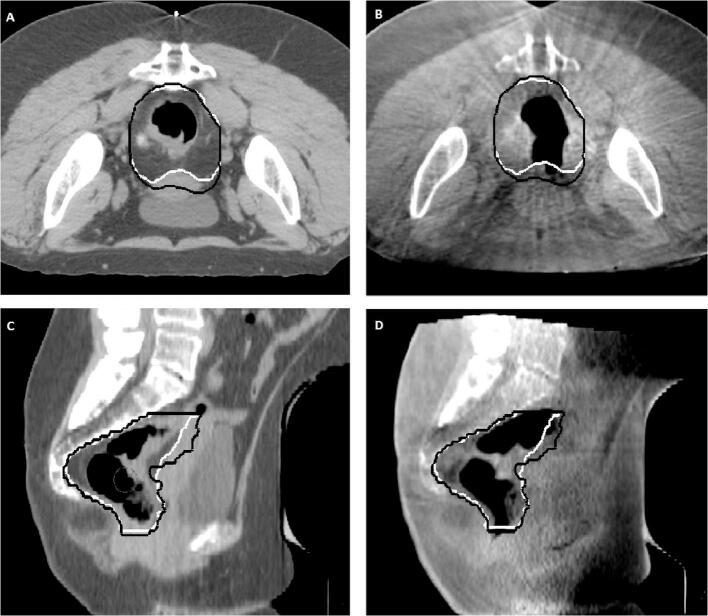
Delineation of the mesorectal part of the CTV on pCT (A, C) and CBCT (B, D) for a female patient. Both transversal (A, B) and sagittal view (C, D) are shown. White and black lines indicate the target delineation according to pCT and CBCT, respectively.

On each pCT and CBCT scan the mesorectal part of the CTV was delineated (Fig. 1); no contrast CT nor MRI were used. The anterior, posterior and lateral borders of the mesorectum were defined by the mesorectal fascia, pelvic muscles and sacrum. To compare both upper and lower mesorectal shape variation, the mesorectum was delineated along the entire craniocaudal axis, ranging from the anorectal junction up to the rectosigmoid junction. The anorectal junction was defined at the level of the insertion of the levator ani muscle into the external sphincter muscles (i.e., disappearing of the mesorectal fat around the rectum) [21]. The rectosigmoid junction was defined using the sigmoid take-off as an anatomical landmark [22]. A horizontal line through the apex of the os sacrum was used to divide upper and lower mesorectum. The lymph nodes regions of the CTV were delineated on each pCT scan according to international consensus guidelines [21]. The CBCT scans were registered to the pCT scan on bony anatomy (converted to translations only) in XVI (Elekta, Crawley, UK). Then, the CBCT delineations were performed in the treatment planning system (TPS) Pinnacle (Philips Medical Systems, Fitchburg, USA). During delineation on the CBCT scan, the pCT delineation was available to guide the observer. All scans were delineated by one observer and evaluated by at least one experienced radiation oncologist.

### 2.3 Mesorectal shape variation

**Fig. 2:**
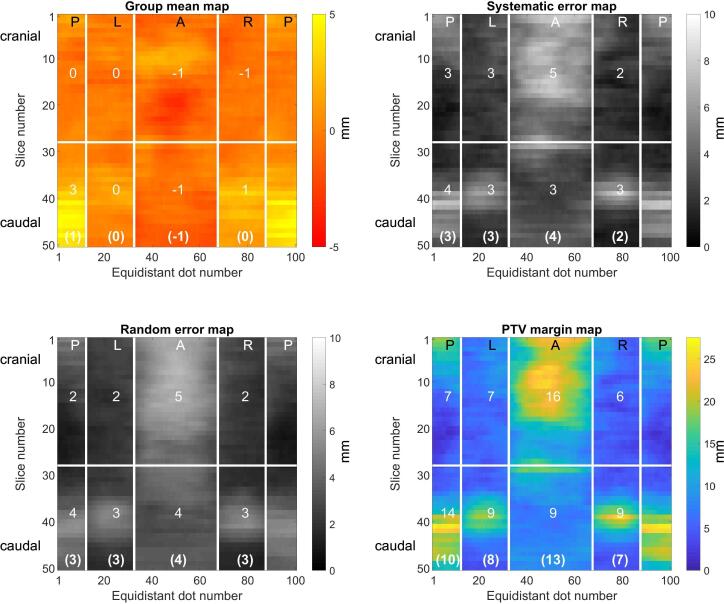
Local group mean (GM), systematic- (∑), random error (σ) and PTV margin for the total group of patients. The horizontal axis represents the 100 equidistant points of each slice starting at posterior (P), via left (L), anterior (A), right (R) and back to posterior. The vertical axis represents the 50 slices ordered from cranial to caudal. The horizontal line indicates the mean slice number of the apex of the os sacrum. The bold numbers in parentheses at the bottom represent the combined outcomes of the upper and lower mesorectum for each direction.

The analysis of mesorectal shape variation was similar to the method described by Nijkamp et al. [17]. The mesorectum delineations of all scans were interpolated along the craniocaudal axis to 50 slices, independent on the length of the mesorectum. On each slice, 100 equidistant points were placed, starting at the posterior side of the patient via left, anterior, right and back to posterior. As the first point on each slice, the point closest to the midline between the leftmost and rightmost point at the posterior side was chosen. This is a reproducible anatomical point, giving a consistent distribution of the 100 points between slices. For each of the 50 × 100 points of each CBCT scan, the signed 1D distance in lateral (x) or anterior-posterior (y) direction was calculated between corresponding points on the pCT and the CBCT delineations. For points on the left/right side the distance was measured in lateral (x) direction and for the anterior/posterior side in anterior-posterior (y) direction; the subdivision of the 50 × 100 points in anterior, left, posterior and right subregions is indicated in Fig. 2. For each patient the mean and standard deviation (SD) were calculated over the five CBCT scans for each point and stored in 2D surface maps. Subsequently, the local group mean (GM), systematic (∑) and random error (σ) maps of the total group were calculated by the mean of the means, the SD over the means, and the root-mean-square of the SDs, respectively. The local means were checked for normality (Lilliefors test, significance level p < 0.05). The local GM was defined positive when the mean of the means of the CBCT positions was distal (in x direction for left/right and in y direction for anterior/posterior) to the CT position and negative otherwise. Data analyses were performed using Matlab (MathWorks, Natick, USA).

### 2.4 PTV margins

To derive PTV margins m_PTV_ for the mesorectum, we used the recipe of Nijkamp et al. [14], i.e., m_PTV_ = α *∑* + β √(*σ*^2^ + σ_p_) – β σ_p_ + GM. ∑ and σ are the SDs of the systematic and random errors, respectively; σ_p_ is the SD describing the penumbra (σ_p_ = 3.2 mm, corresponding to water and suitable for the pelvic region). The additional term GM was added by Nijkamp et al. [14] to the original formula of van Herk et al. [23] to include deformations, taking the margin direction into account. To ensure a minimum CTV dose of 95% of the prescribed dose for at least 90% of the patients, α = 2.15 and β = 1.64 were used; note that α was different from the mostly used value of 2.5, because here the margins were effectively analyzed in 2D [23].

### 2.5 Statistical analysis

Four different groups were compared, being male vs. female, and RT vs. CRT patients. For the GM and the σ maps, comparison between groups was done using the Wilcoxon rank sum test. The ∑ maps were compared between groups using a two-sided F-test. For all comparisons statistical significance level was set to p < 0.05.

### 2.6 Dose coverage

A simulation was performed to test if the total PTV, based on the mesorectal and lymph node CTVs and the clinically used PTV margins, was sufficient for the mesorectal shape variation throughout treatment. For each patient a total PTV was constructed using the clinical margins for the mesorectal CTV (15 mm in anterior direction and 10 mm in all other directions) and the clinical margins for the lymph node regions (10 mm in all directions). Then, an approximation of an ideal dose distribution was created where the 95% isodose surface enclosed the total PTV. This was done by expanding the total PTV by β σ_p_ = 1.64 × 3.2 mm = 5.2 mm in three dimensions in the TPS and then convolving the resulting 3D surface with a 3D Gaussian with σ_p_ = 3.2 mm. Finally, for each patient, the dose was sampled in this ideal dose distribution for all 50 × 100 points of the mesorectal CTV delineated on each CBCT scan, and summed per point to get the cumulative dose on the mesorectal CTV. The dose coverage of the mesorectal CTV was calculated as the percentage of points with a dose ≥ 95% of the prescription dose.

## 3 Results

The null hypothesis for normality for the local 50 × 100 mean values over the total patient group was not rejected for the majority of points (84%). GM variation in the whole patient group was relatively small, ranging between −1 mm in the anterior region and 3 mm in the lower-posterior region (Fig. 2). ∑ and σ were ranging from 2 mm in the upper-lateral region up to 5 mm in the upper-anterior region. For the whole group, there was no significant difference for GM, ∑ and σ between the left and right region; therefore, left and right were combined to “lateral” for further statistical analysis. The only significant differences found were between male and female patients for the GM of the lower-lateral subregions of 1.8 mm and between RT and CRT patients for σ of the anterior region of approximately 1.1 mm.

Derived PTV margins were smallest in the upper-lateral region (6 mm) and largest in the upper-anterior region (16 mm), as depicted in Fig. 2. Differences between subgroups larger than 3 mm were found for the PTV margins for the upper-anterior region, which were larger for female (19 mm) compared to male patients (14 mm). Margins were comparable between RT and CRT patients (Table 2). For the whole group of patients for the whole length of the mesorectal CTV, the margins for mesorectal shape variations were on average 13 mm anteriorly, 10 mm posteriorly and 7–8 mm laterally (Fig. 2). The dose coverage was better than 99% for all patients but one, where coverage was 97%.

**Table 2:** PTV margins (in mm) according to gender and radiotherapy course.

	Male				Female			
	Anterior	Posterior	Left	Right	Anterior	Posterior	Left	Right
Upper mesorectum	14	7	5	4	19	8	8	7
Lower mesorectum	8	12	7	7	8	14	10	10
Total mesorectum	11	9	6	5	14	11	9	8

	5 × 5 Gy				25 × 2 Gy			

	Anterior	Posterior	Left	Right	Anterior	Posterior	Left	Right

Upper mesorectum	15	9	7	6	17	6	6	5
Lower mesorectum	8	15	10	9	9	12	7	8
Total mesorectum	12	12	8	8	14	9	6	6

The apex of the os sacrum was chosen as anatomical border to divide upper and lower mesorectum.

## 4 Discussion

The current study showed that mesorectal shape variation is heterogeneous, in rectal cancer patients irradiated in prone position using a belly board. Our clinical margins for the total PTV were sufficient to encompass mesorectal shape variations.

Literature focusing on inter-fraction mesorectal shape variation in rectal cancer irradiation is scarce. In most studies patients were irradiated either in supine position or prone position without using a belly board [14], [16], [17], [18], [19], [20]. Tournel et al. described mesorectal shape variation in ten CRT patients placed in prone position without use of a belly board, with daily CT scanning [19]. A mean mesorectal shift of −2 mm (1SD = 6.8 mm) and −0.4 mm (1SD = 3.8 mm) was found in anterior and posterior direction, respectively. However, these results were averaged over the craniocaudal axis and over all patients, ignoring the heterogeneity of shape variation and the influence of gender. Nijkamp et al. evaluated CTV motion for 63 rectal cancer patients in supine position using repeat CT imaging [14]. The random error was found 4–7 mm, which is in line with our results. Systematic errors were ranging from 2 mm close to bony structures up to 10 mm at the upper-anterior part of the mesorectum. In our study a maximum systematic error of 6 mm at the upper-anterior region was found in the female patient group (data not shown). This difference in maximum systematic error might be explained by the use of a belly board, different bladder filling protocols, variation in target delineation and/or tumor location. Cranmer-Sargison et al. described a PTV margin evaluation for 24 rectal cancer patients irradiated in prone position using a belly board. The overall difference in mean CTV position was 1.1 mm (1SD = 0.4 mm) and 0.0 mm (1SD = 0.2 mm) in anterior-posterior and left–right direction, respectively. They used 3D bony anatomy matching between pCT and CBCT as a surrogate for inter-fractional CTV positional errors. Therefore, they assumed an equivalence between CTV and pelvic bony structure position. However, this is not the case for mesorectal shape variation in anterior direction, as the MRF there is not confined by pelvis muscle/bone. This approach may result in an underestimation of actual inter-fraction CTV shape variation, especially regarding the mesorectum in anterior direction.

In order to calculate PTV margins, the margin recipe of Nijkamp et al. was adapted to a 2D situation [14]. According to van Herk et al., we used α = 2.15 for the impact of systematic errors [23]. Multiple values have been used for α, for example α = 1.96 and α = 3.2, which apply to a 1D and 3D situation, respectively. As a result of this, PTV margins derived by Nijkamp et al. (α = 3.2) were larger compared to our margins [14]. Cranmer-Sargison et al. used α = 1.96 with PTV margins of 7.0 mm in anterior-posterior and 5.0 mm in left–right direction, including correction for intra-fractional CTV motion up to 3.0 mm [15]. In anterior direction, our current PTV margin for the mesorectal CTV is 15 mm along the entire craniocaudal axis. For the lower mesorectum we calculated a (average) margin of 9 mm in anterior direction, which was comparable for male and female patients. Therefore, it seems safe to reduce the PTV margin for the lower-anterior region to 10 mm. The calculated margin for the upper-anterior region was larger in the female patient group (19 mm) compared to the male patient group (14 mm). Minimal differences in GM, ∑ or σ (maximum 1 mm SD) were found between both groups. It is probably the combination of these three components in the margin recipe that accounts for the larger PTV margin in the female patient group. These results are in line with Nijkamp et al., who also suggested larger margins are required in the upper-anterior region for female patients treated in prone position [17]. This can possibly be explained by gender-related differences in anatomy. MRI-based studies in patients with cervical cancer demonstrated that the uterus shape and position can change several centimeters from day-to-day [24], [25].

PTV margins for the mesorectal CTV in this study were developed based on averages of the local margins in several regions. As a consequence, the coverage of the mesorectal CTV will only be correct for the *average* of all points and certain parts of the CTV will be underdosed. To get 95% minimum dose for the mesorectal CTV, the *maximum* value of each region should be used, resulting in very large margins. However, clinically, those large margins are not used to our knowledge. In rectal cancer radiotherapy also the lymph node regions have to be irradiated [21]. When the lymph node regions were included in the total PTV, the resulting PTV was large enough to “buffer” the shape variations of the mesorectum, although the margins of the mesorectal CTV on its own were in principle to small for the entire mesorectum. This could be shown using an approximation of an ideal dose distribution.

This study had several limitations. First, the nodal subregions of the CTV were not delineated, because low inter-fraction shape variation was expected and these structures are difficult to delineate on low resolution CBCT scans. Nijkamp et al. are the only group that investigated shape variation of the internal iliac, obturatorial and presacral lymph node regions, describing PTV margins ranging from 7 mm in posterior direction to 15 mm in anterior direction [14]. Second, mesorectal shape variation was assessed in a 2D situation and was not evaluated in craniocaudal direction. Third, delineation variation, intra-fraction setup errors and intra-fraction mesorectal shape variation were not investigated and thus not included in the margin calculation. Delineation variation was minimized by having one observer for all patients, availability of the pCT delineations during CBCT delineation, and evaluation of all delineations together with one of the two selected radiation oncologists. Because of the time scale, intra-fraction mesorectal shape variations are not larger than the inter-fraction variations, and were thus indirectly taken care of in our analysis. Whereas inter-fraction setup errors were addressed by online corrections based on bony anatomy, intra-fraction errors also contribute to the overall uncertainty. Nijkamp et al. reported intra-fraction setup errors up to 2.4 mm in left–right direction, regarding prone positioning without belly board [17]. Also other uncertainties, such as registration uncertainties, residual rotations, accelerator-related mechanical uncertainties should be taken into account in the total PTV margins. As this is a complex process, end-to-end tests can be used for estimates [26]; due to their size in the order of millimeters, the impact of these uncertainties is limited.

In conclusion, this study demonstrated that mesorectal shape variation is heterogeneous and largest in the upper-anterior region, in rectal cancer patients irradiated in prone position with the use of a belly board. Calculated PTV margins were ranging from 6 mm in the upper-lateral region up to 16 mm in the upper-anterior region of the mesorectum. Subgroup analysis revealed that a larger PTV margin in the upper-anterior region is needed for female patients compared to male patients. In literature, a large variety of PTV margins has been reported for both prone and supine positioning, especially in the anterior direction. Therefore, it remains important to determine institutional PTV margins depending on patient positioning, radiation technique and online imaging.

## Declaration of Competing Interest

The authors declare that they have no known competing financial interests or personal relationships that could have appeared to influence the work reported in this paper.

## References

[1] Heald R.J., Ryall R.D.H. (1986). Recurrence and survival after total mesorectal excision for rectal cancer. Lancet.

[2] National Institute for Clinical Excellence. Colorectal Cancer. NICE guideline [NG151], https://www.nice.org.uk/guidance/ng151/; 2020 [accessed 18 May 2020]. 32813481

[3] MacFarlane J.K., Ryall R.D.H., Heald R.J. (1993). Mesorectal excision for rectal cancer. Lancet.

[4] van Gijn W., Marijnen C.AM., Nagtegaal I.D., Kranenbarg E.-K., Putter H., Wiggers T. (2011). Preoperative radiotherapy combined with total mesorectal excision for resectable rectal cancer: 12-year follow-up of the multicentre, randomised controlled TME trial. Lancet Oncol.

[5] Sauer R., Becker H., Hohenberger W., Rödel C., Wittekind C., Fietkau R. (2004). Preoperative versus postoperative chemoradiotherapy for rectal cancer. N Engl J Med.

[6] Sauer R., Liersch T., Merkel S., Fietkau R., Hohenberger W., Hess C. (2012). Preoperative versus postoperative chemoradiotherapy for locally advanced rectal cancer: results of the German CAO/ARO/AIO-94 randomized phase III trial after a median follow-up of 11 years. J Clin Oncol.

[7] Minsky B.D., Conti J.A., Huang Y., Knopf K. (1995). Relationship of acute gastrointestinal toxicity and the volume of irradiated small bowel in patients receiving combined modality therapy for rectal cancer. J Clin Oncol.

[8] Letschert J.G.J., Lebesque J.V., de Boer R.W., A.M. Hart A., Bartelink H. (1990). Dose-volume correlation in radiation-related late small-bowel complications: a clinical study. Radiother Oncol.

[9] Wiesendanger-Wittmer E.M., Sijtsema N.M., Muijs C.T., Beukema J.C. (2012). Systematic review of the role of a belly board device in radiotherapy delivery in patients with pelvic malignancies. Radiother Oncol.

[10] Kim T.H., Chie E.K., Kim D.Y., Park S.Y., Cho K.H., Jung K.H. (2005). Comparison of the belly board device method and the distended bladder method for reducing irradiated small bowel volumes in preoperative radiotherapy of rectal cancer patients. Int J Radiat Oncol Biol Phys.

[11] Dröge L.H., Weber H.E., Guhlich M., Leu M., Conradi L.-C., Gaedcke J. (2015). Reduced toxicity in the treatment of locally advanced rectal cancer: a comparison of volumetric modulated arc therapy and 3D conformal radiotherapy. BMC Cancer.

[12] Wee CW, Kang HC, Wu HG, Chie EK, Choi N, Park JM, et al. Intensity-modulated radiotherapy versus three-dimensional conformal radiotherapy in rectal cancer treated with neoadjuvant concurrent chemoradiation: a meta-analysis and pooled-analysis of acute toxicity. Jpn J Clin Oncol. 2018;48:458-66. https://doi.org/10.1093/jjco/hyy029. 10.1093/jjco/hyy029 29554287

[13] Nuyttens J.J., Robertson J.M., Yan D.i., Martinez A. (2002). The variability of the clinical target volume for rectal cancer due to internal organ motion during adjuvant treatment. Int J Radiat Oncol Biol Phys.

[14] Nijkamp J., Swellengrebel M., Hollmann B., de Jong R., Marijnen C., van Vliet-Vroegindeweij C. (2012). Repeat CT assessed CTV variation and PTV margins for short- and long-course pre-operative RT of rectal cancer. Radiother Oncol.

[15] Cranmer-Sargison G., Kundapur V., Park-Somers E., Andreas J., Vachhrajani H., Sidhu N.P. (2013). Planning target volume margin evaluation and critical structure sparing for rectal cancer patients treated prone on a bellyboard. Clin Oncol (R Coll Radiol).

[16] Brierley J.D., Dawson L.A., Sampson E., Bayley A., Scott S., Moseley J.L. (2011). Rectal motion in patients receiving preoperative radiotherapy for carcinoma of the rectum. Int J Radiat Oncol Biol Phys.

[17] Nijkamp J., de Jong R., Sonke J.-J., Remeijer P., van Vliet C., Marijnen C. (2009). Target volume shape variation during hypo-fractionated preoperative irradiation of rectal cancer patients. Radiother Oncol.

[18] Bondar L., Intven M., Burbach J.P., Budiarto E., Kleijnen J.P., Philippens M. (2014). Statistical modeling of CTV motion and deformation for IMRT of early-stage rectal cancer. Int J Radiat Oncol Biol Phys.

[19] Tournel K., De Ridder M., Engels B., Bijdekerke P., Fierens Y., Duchateau M. (2008). Assessment of intrafractional movement and internal motion in radiotherapy of rectal cancer using megavoltage computed tomography. Int J Radiat Oncol Biol Phys.

[20] Nijkamp J., de Jong R., Sonke J.-J., van Vliet C., Marijnen C. (2009). Target volume shape variation during irradiation of rectal cancer patients in supine position: comparison with prone position. Radiother Oncol.

[21] Valentini V., Gambacorta M.A., Barbaro B., Chiloiro G., Coco C., Das P. (2016). International consensus guidelines on Clinical Target Volume delineation in rectal cancer. Radiother Oncol.

[22] D'Souza N., de Neree tot Babberich M.P.M., Lord A., Shaw A., Abulafi M., Tekkis P. (2018). The rectosigmoid problem. Surg Oncol.

[23] van Herk M., Remeijer P., Rasch C., Lebesque J.V. (2000). The probability of correct target dosage: dose-population histograms for deriving treatment margins in radiotherapy. Int J Radiat Oncol Biol Phys.

[24] Taylor A., Powell M.E. (2008). An assessment of interfractional uterine and cervical motion: implications for radiotherapy target volume definition in gynaecological cancer. Radiother Oncol.

[25] van de Bunt L., Jürgenliemk-Schulz I.M., de Kort G.A.P., Roesink J.M., Tersteeg R.J.H.A., van der Heide U.A. (2008). Motion and deformation of the target volumes during IMRT for cervical cancer: what margins do we need?. Radiother Oncol.

[26] Seravalli E., van Haaren P.M.A., van der Toorn P.P., Hurkmans C.W. (2015). A comprehensive evaluation of treatment accuracy, including end-to-end tests and clinical data, applied to intracranial stereotactic radiotherapy. Radiother Oncol.

